# Beneficial Effects of Transplanted Human Bone Marrow Endothelial Progenitors on Functional and Cellular Components of Blood-Spinal Cord Barrier in ALS Mice

**DOI:** 10.1523/ENEURO.0314-21.2021

**Published:** 2021-09-16

**Authors:** Svitlana Garbuzova-Davis, Kayla J. Boccio, Alexander Llauget, Robert Shell, Surafuale Hailu, Hilmi Mustafa, Jared Ehrhart, Paul R. Sanberg, Stanley H. Appel, Cesario V. Borlongan

**Affiliations:** 1 Center of Excellence for Aging and Brain Repair, University of South Florida, Morsani College of Medicine, Tampa, FL 33612; 2 Department of Neurosurgery and Brain Repair, University of South Florida, Morsani College of Medicine, Tampa, FL 33612; 3 Department of Molecular Pharmacology and Physiology, University of South Florida, Morsani College of Medicine, Tampa, FL 33612; 4 Department of Pathology and Cell Biology, University of South Florida, Morsani College of Medicine, Tampa, FL 33612; 5 Department of Psychiatry, University of South Florida, Morsani College of Medicine, Tampa, FL 33612; 6 Stanley H. Appel Department of Neurology, Houston Methodist Neurological Institute, Houston, TX 77030

**Keywords:** ALS, blood-spinal cord barrier, G93A SOD1 mice, human bone marrow-derived stem cells, repair, transplantation

## Abstract

Convincing evidence of blood-spinal cord barrier (BSCB) alterations has been demonstrated in amyotrophic lateral sclerosis (ALS) and barrier repair is imperative to prevent motor neuron dysfunction. We showed benefits of human bone marrow-derived CD34+ cells (hBM34+) and endothelial progenitor cells (hBM-EPCs) intravenous transplantation into symptomatic G93A SOD1 mutant mice on barrier reparative processes. These gains likely occurred by replacement of damaged endothelial cells, prolonging motor neuron survival. However, additional investigations are needed to confirm the effects of administered cells on integrity of the microvascular endothelium. The aim of this study was to determine tight junction protein levels, capillary pericyte coverage, microvascular basement membrane, and endothelial filamentous actin (F-actin) status in spinal cord capillaries of G93A SOD1 mutant mice treated with human bone marrow-derived stem cells. Tight junction proteins were detected in the spinal cords of cell-treated versus non-treated mice via Western blotting at four weeks after transplant. Capillary pericyte, basement membrane laminin, and endothelial F-actin magnitudes were determined in cervical/lumbar spinal cord tissues in ALS mice, including controls, by immunohistochemistry and fluorescent staining. Results showed that cell-treated versus media-treated ALS mice substantially increased tight junction protein levels, capillary pericyte coverage, basement membrane laminin immunoexpressions, and endothelial cytoskeletal F-actin fluorescent expressions. The greatest benefits were detected in mice receiving hBM-EPCs versus hBM34+ cells. These study results support treatment with a specific cell type derived from human bone marrow toward BSCB repair in ALS. Thus, hBM-EPCs may be advanced for clinical applications as a cell-specific approach for ALS therapy through restored barrier integrity.

## Significance Statement

Repair of the disease-altered blood-spinal cord barrier (BSCB) in amyotrophic lateral sclerosis (ALS) via cell transplantation may be a feasible therapeutic approach. We showed benefits of intravenously transplanted human bone marrow-derived stem cells into symptomatic G93A SOD1 mutant mice on barrier reparative processes, likely by replacing damaged endothelial cells. However, effects of administered cells on endothelium integrity in mouse CNS capillaries was not fully determined. Here, we showed that ALS mice receiving human bone marrow endothelial progenitor cells (hBM-EPCs) versus hBM34+ cells substantially increased tight junction protein levels, capillary pericyte coverage, basement membrane laminin immunoexpressions, and endothelial cytoskeletal filamentous actin (F-actin) fluorescent expressions. These results provide evidence that hBM-EPCs effectively maintained capillary endothelium integrity in ALS and these cells may be advanced for clinical applications restoring BSCB integrity.

## Introduction

Amyotrophic lateral sclerosis (ALS) is characterized by motor neuron degeneration in the brain and spinal cord leading to paralysis and fatality within three to five years of diagnosis (Cleveland and Rothstein, 2001; Lomen-Hoerth, 2008; Talbot, 2009; Kiernan et al., 2011). Treatment options are mainly supportive and therapy development is complicated by diffuse motor neuron degeneration in the CNS and by complex factors underlying disease pathogenesis (Martin et al., 2000; Strong et al., 2005; Rothstein, 2009; Wijesekera and Leigh, 2009).

Our original studies (Garbuzova-Davis et al., 2007a, b) demonstrated structural and functional disruptions of the blood-brain barrier (BBB) and blood-spinal cord barrier (BSCB) in symptomatic G93A SOD1 mutant mice. Later reports provided evidence of barrier alterations in animal disease models before motor neuron degeneration (Zhong et al., 2008; Nicaise et al., 2009; Miyazaki et al., 2011). These studies showed alterations of capillary endothelial cells (ECs), astrocyte end-feet processes, and tight junction protein levels. Also, in postmortem CNS tissues from ALS patients, dysfunctional capillary endothelial cells, downregulated tight junction proteins, reduced pericyte capillary coverage, and microvascular leakage were determined (Henkel et al., 2009; Garbuzova-Davis et al., 2012; Winkler et al., 2013; Sasaki, 2015; Yamadera et al., 2015). BBB/BSCB damage may identify ALS as a neurovascular disease (Garbuzova-Davis et al., 2011; Rodrigues et al., 2012). Moreover, molecular disease biomarkers were discussed (Wolburg et al., 2009; Nicaise et al., 2010; Bataveljic et al., 2014). Thus, barrier impairment could aggravate ALS motor neuron degeneration and even trigger disease related pathologies (Garbuzova-Davis et al., 2008; Kakaroubas et al., 2019). Since CNS vascular homeostasis is disrupted in ALS, barrier restoration via cell administration may be a feasible therapeutic approach.

We investigated potential endothelium repair in ALS by intravenous (iv) stem cell delivery into symptomatic G93A SOD1 mice. Different doses of human bone marrow-derived CD34+ cells (hBM34+) were iv administered into ALS mice. Results, primarily from the highest dose of 1 × 10^6^ cells/mouse, showed decreased capillary permeability, restored capillary ultrastructure, reduced macrogliosis and microgliosis, decreased microhemorrhages, and enhanced motor neuron survival in the spinal cord (Garbuzova-Davis et al., 2017b, 2018; Eve et al., 2018). However, a delayed effect on motor function and still severely damaged capillaries were observed in hBM34+ posttransplanted ALS mice. In our follow-up study, human bone marrow-derived endothelial progenitor cells (hBM-EPCs, 1 × 10^6^ cells/mouse) were iv transplanted into symptomatic G93A SOD1 mutants. Cells significantly improved behavioral outcomes leading to delayed disease progression (Garbuzova-Davis et al., 2019b). This likely occurred because of widespread distribution of transplanted cells and their engraftment into CNS capillary lumen, restoring capillary ultrastructure, decreasing capillary permeability, and maintaining motor neuron survival. Results demonstrated that systemic administration of hBM-EPCs versus hBM34+ cells into symptomatic ALS mice effectively restored CNS barrier integrity, potentially by replacing damaged ECs.

However, effects of transplanted hBM34+ cells or hBM-EPCs on “tightness” between ECs, pericytes, and basement membrane status were not addressed. Also, EC integrity within capillaries in mouse CNS tissues was not fully determined. One indicator of cellular function is the presence of microfilaments, proteins for cytosolic organelle organization (Fletcher and Mullins, 2010; Dominguez and Holmes, 2011). Filamentous (F-actin) stabilizes morphologic cellular homeostasis. We showed re-arrangement of F-actin within hBM-EPCs *in vitro* under normogenic conditions (Garbuzova-Davis et al., 2019a). Cultured cells displayed re-organized strips of F-actin in cytosol, supporting the importance of cytoskeletal actin during EC proliferation and motility (Waschke et al., 2005; Kozuka et al., 2007; Stricker et al., 2010). Also, ZO-1 and occludin levels in hBM-EPCs co-localized with F-actin filaments *in vitro*, evidencing intracellular integrity and connectivity. Although involvement of F-actin in ALS is unclear, potential dysregulation of the actin cytoskeleton in association with the actin-binding protein profilin-1 has been discussed (Hensel and Claus, 2018).

The aim of this study was to determine tight junction protein levels, capillary pericyte coverage, and microvascular basement membrane status in spinal cords of G93A SOD1 mutant mice treated with human bone marrow stem cells. A specific focus was examining endothelial F-actin.

## Materials and Methods

### Experimental design and statistical analyses

#### Ethics statement

All described procedures were approved by the Institutional Animal Care and Use Committee at University of South Florida and conducted in compliance with the *Guide for the Care and Use of Laboratory Animals*. All mice were housed in a temperature-controlled room (23°C) and maintained on a 12/12 h light/dark cycle (lights on at 6 A.M.). Food and water were available *ad libitum*. Upon progression of neurologic symptoms, a highly palatable liquid nutritional supplement was placed on the cage floor, ensuring access by the animal.

#### Animals

All animals were obtained from The Jackson Laboratory. Forty-eight transgenic male B6SJL-Tg(SOD1*G93A)1Gur/J mice, over-expressing human SOD1 carrying the Gly93→Ala mutation (G93A SOD1) at seven weeks of age, were randomly assigned to one of three groups receiving human bone marrow stem cells or media: group 1, hBM34+ cells (1 × 10^6^ cells/mouse, *n* = 15); group 2, hBM-EPCs (1 × 10^6^ cells/mouse, *n* = 15); and group 3, media (*n* = 18). ALS mice intravenously (jugular vein) were administered with hBM34+ cells, hBM-EPCs, or the same volume of media at 13 weeks of age, an early symptomatic disease stage. Group 4 mice, non-transplant controls (*n* = 17), were animals from the background strain not carrying the mutant SOD1 gene.

#### Cell preparation and transplant procedure

Cryopreserved human bone marrow CD34+ cells (hBM34+) and human bone marrow-derived endothelial progenitor cells (hBM-EPCs) were purchased from AllCells and CELPROGEN, respectively. According to the company reports, cells were obtained from healthy adult donors and tested negative for various viruses and microbial growth using an infectious disease panel.

Detailed preparation of hBM34+ cells or hBM-EPCs for transplantation was previously described in our reports (Garbuzova-Davis et al., 2017b, 2018, 2019b). Briefly, cells were thawed rapidly at 37°C and then transferred into a centrifuge tube containing 12 ml of Dulbecco’s PBS 1× (DPBS), pH 7.4 (Mediatech). The cells were centrifuged (hBM34+: 200 × *g*/10 min, hBM-EPCs: 100 × *g*/7 min) at room temperature (RT), the supernatant discarded, and the process repeated. After the final wash, cell viability was assessed using the 0.4% trypan blue dye exclusion method before transplantation. Cell concentration was adjusted to 5000 cells/μl (1 × 10^6^ cells/200 μl/injection).

The hBM34+ cells or hBM-EPCs were delivered intravenously via the jugular vein of mice under anesthesia with isofluorane (2–5% at 2 l O_2_/min) as we previously described (Garbuzova-Davis et al., 2017b, 2018, 2019b). The media mice in group 3 received 200 μl of DPBS, the same volume administered to the cell-transplanted mice. Animals in groups 1–3 received cyclosporine A (CsA; 10 mg/kg, i.p.) daily for the entire posttransplant period.

#### Perfusion and tissue preparation

All cell-treated, media-treated, and control mice were killed at 17 weeks of age (four weeks after cell or media administration) for tight junction protein levels via Western blottings and immunohistochemical analyses of the spinal cords. Briefly, animals were killed under Euthasol (0.22 ml/kg body mass) and perfused transcardially with 0.1 m phosphate buffer (PB; pH 7.2) using our previously described perfusion techniques (Garbuzova-Davis et al., 2017b, 2018, 2019b). Mice (*n* = 8/group) assayed for Western blotting received only PB and on perfusion, the entire spinal cords were rapidly removed from hBM34+ cell-treated, hBM-EPC-treated, media-treated, and controls, weighed, placed in cryovials, and stored at −80°C for further examination of tight junction protein levels. In the remaining mice (*n* = 4–5/group), after perfusion with PB and followed by 4% paraformaldehyde (PFA) in PB solution, segments of cervical and lumbar spinal cord were removed, postfixed in 4% PFA for 24–48 h, and then cryopreserved in 20% sucrose in 0.1 m PB overnight. Coronal spinal cord tissues were cut at 30-μm thickness by a cryostat and every fifth section was thaw-mounted onto slides. Collected spinal cord tissues were stored at −20°C for further immunohistochemical analyses of capillary pericyte coverage and endothelial F-actin.

#### Western blotting

Detection of tight junction (ZO-1, occludin, and claudin-5) proteins in the spinal cords of cell-treated, media-treated ALS mice, and controls (*n* = 8/group) was performed via Western blot analysis similarly to our pervious report (Garbuzova-Davis et al., 2012). Briefly, the spinal cord tissues obtained from hBM34+ cell-treated, hBM-EPC-treated, media-treated, and control mice were briefly homogenized and sonicated in ice-cold 2× cell lysis buffer (catalog #9803, Cell Signaling Technology) with 1% protease/phosphate inhibitor cocktail [Halt Protease and Phosphate Inhibitor Single-Use Cocktail (100×), catalog #78442, ThermoFisher Scientific]. Tissues were lysed for 1 h on ice and then centrifuged at 14,000 rpm for 15 min at 4°C. The supernatant was aliquoted in 40 μl samples and stored at −80°C. Total protein concentration was determined by protein assay (Pierce BCA Protein Assay kit, catalog #23277, ThermoFisher Scientific). Samples were denatured at 70°C for 8 min in Laemmli sample buffer (catalog #1610737, Bio-Rad) with 5% β-mercaptoethanol (2-mercaptoethanol, catalog #1610710, Bio-Rad). Electrophoresis was performed using mini-protean gels (4–15% Mini-PROTEAN TGX, catalog #4561086, Bio-Rad) loaded with 20–25 μg of total protein per sample/well and 40V was applied for 30 min followed by 90 V for 45 min. Proteins were transferred to nitrocellulose membranes (Nitrocellulose Membrane Roll, 0.2 μm, catalog #1620112, Bio-Rad) using Criterion Blotter (Bio-Rad) for blotting efficiency and then 90V was applied for 45 min. The membranes were blocked in 5% non-fat dry milk (Blotting-Grade Blocker, catalog #1706404, Bio-Rad) in 1× Tris-buffered saline (10× TBS; catalog #1706435, Bio-Rad) for 1 h. Membranes were then incubated overnight at 4°C with goat polyclonal *anti-GAPDH* (1:2000, catalog #ab157156, Abcam) and one of the following primary antibodies: rabbit polyclonal *anti-ZO-1* (1:2000, catalog #617300, ThermoFisher Scientific), rabbit monoclonal *anti-occludin* (1:500, catalog #ab224526, Abcam), or rabbit polyclonal *anti-claudin-5* (1:1000, catalog #ab15106, Abcam). The next day, membranes were washed with 1× TBS buffer (catalog #1706435, Bio-Rad) with 0.1% Tween 20 (catalog #P1379, Sigma-Aldrich) and then incubated with one of the following secondary antibodies: IRDye 800CW (green) donkey-anti-rabbit IgG (1:10,000, catalog #92632213, Li-COR) or IRDye 680LT (red) donkey-anti-goat IgG (1:30,000, catalog #92668024, Li-COR) for 2 h at RT. After incubation, the membranes were washed with 0.1% Tween/TBST buffer followed by 1× TBS three times for 10 min each and then scanned on a Li-COR Odyssey^R^ Infrared Imaging System (model 9120, version 3.0) to measure infrared (IR) signal. Images were analyzed using Li-COR Image Studio 4.0 software and band signals measured at 700/800 wavelength. For each evaluated protein, the signal was normalized to GAPDH signal. Fold IR signal changes relative to controls were calculated.

#### Immunohistochemistry and fluorescent staining

For identification of capillary pericytes, serial cervical and lumbar spinal cord tissue sections from randomly selected cell-treated, media-injected, and control mice (*n* = 4–5/group) were immunostained with appropriate primary and secondary antibodies. For double immunostaining of capillary pericyte and laminin, tissue sections were thawed and preincubated with 10% normal goat serum (NGS) and 3% Triton 100X in PBS for 60 min at RT. Afterwards, rat monoclonal anti-CD13 (1:50, catalog #MA1-33449, Invitrogen) primary antibody was applied on tissues overnight at 4°C. After rinsing slides in PBS, tissues were incubated with goat anti-rat secondary antibody conjugated to rhodamine (1:700, catalog #ab150160, Abcam) for 2 h at RT. The next day, rabbit polyclonal anti-laminin (1:200, catalog #ab11575, Abcam) primary antibody was applied on tissues overnight at 4°C. Then, tissue slides were incubated with goat anti-rabbit secondary antibody conjugated to FITC (1:500, catalog #A11034, Invitrogen) for 2 h at RT. Next, the slides were rinsed in PBS and coverslipped with Vectashield ® containing DAPI (Vector Laboratories). The tissues were examined under epifluorescence using an Olympus BX60 microscope and randomly selected images of ventral horn spinal cord capillaries (*n* = 3–4 mice/group, *n* = 8–15 capillaries/spinal cord segment) were obtained at 40× for further analyses of CD13/laminin fluorescent immunoexpressions.

In a separate set of cervical and lumbar spinal cord sections, double immunostaining for CD31 and fluorescent staining for F-actin were achieved. Initial staining with rat monoclonal anti-CD31 (1:50, catalog #ab56299, Abcam) primary antibody was performed overnight at 4°C after incubation in blocking solution (NGS/Triton/PBS) as described above. Then, tissue slides were incubated with goat anti-rat secondary antibody conjugated to rhodamine (1:700, catalog #ab150160, Abcam) for 2 h at RT. After slides were thoroughly washed in PBS, Acti-Stain 488 fluorescent phalloidin protein (1:150, catalog #SKU PHDG1, Cytoskeleton) was applied on tissues for 60 min at RT. Next, the slides were rinsed in PBS and coverslipped with Vectashield^®^ containing DAPI (Vector Laboratories). The tissues were examined under epifluorescence using an Olympus BX60 microscope and randomly selected images of ventral horn spinal cord capillaries (*n* = 4–5 mice/group, *n* = 8–10 capillaries/spinal cord segment) were obtained at 40× for further analyses of CD31/F-actin expressions.

The antigen expressions were analyzed in cervical and lumbar spinal cord images using NIH ImageJ (version 1.46) software. The image analyses for CD13/laminin and CD31/F-actin were performed by measuring intensity of fluorescent expressions (%/mm^2^) on individual capillaries of ∼20–25 μm. Thresholds for detection of fluorescent antigen expressions were adjusted to each image to eliminate background noise.

#### Statistical analysis

Data are presented as means ± SEM. One-way ANOVA with *post hoc* Tukey’s HSD multiple comparison test using online statistical software (https://astatsa.com/; 2016 Navendu Vasavada) was performed for statistical analysis. Significance was defined as *p* < 0.05.

## Results

### Characteristics of tight junction protein levels in the spinal cord

**Figure 1.**
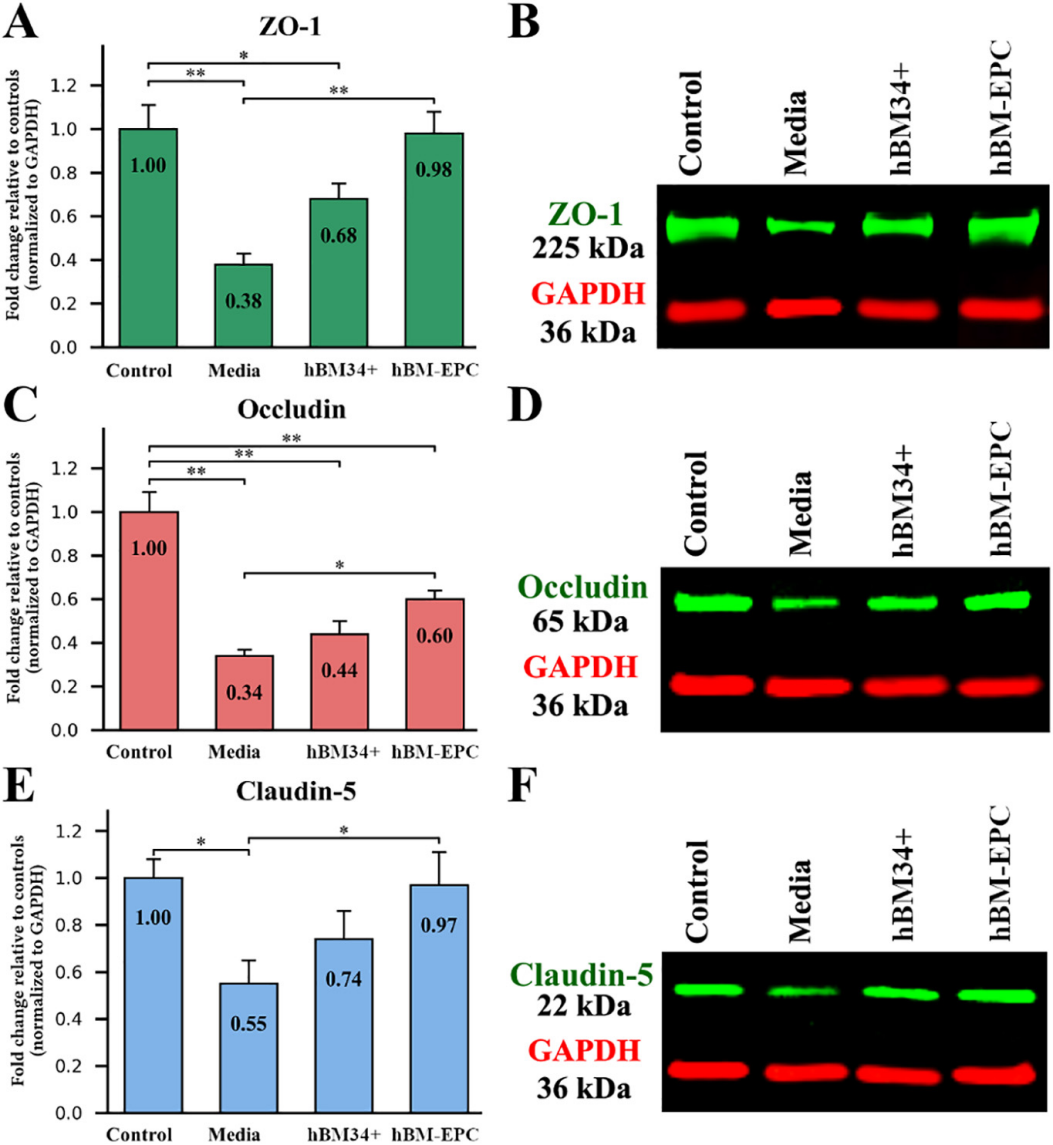
Tight junction protein levels in the spinal cords of G93A SOD1 mice via Western blotting. Media-treated ALS mice showed significant reductions of ZO-1 (***A***, ***B***), occludin (***C***, ***D***), and claudin-5 (***E***, ***F***) protein levels versus controls. Although elevated levels of analyzed tight junction proteins were noted in ALS mice receiving hBM34+ cells, significant differences between media and these cell-treated mice were not found. In contrast, significant increases of ZO-1 (***A***, ***B***), occludin (***C***, ***D***), and claudin-5 (***E***, ***F***) proteins were determined in ALS mice after hBM-EPC transplantation versus media-treated animals. For each evaluated protein (***B***, ***D***, ***F***), the band density was normalized to GAPDH signal and fold signal changes relative to levels of controls were presented; *p < 0.05, **p < 0.01.

Tight junction (ZO-1, occludin, and claudin-5) proteins were analyzed in the spinal cords of cell-treated and media-treated ALS mice in comparison to control animals at 17 weeks of age using Western blotting. For each evaluated protein, the band density was normalized to GAPDH and fold changes relative to levels of controls were presented. Results showed significant downregulation of all analyzed proteins in media-treated ALS mice versus controls: ZO-1 (*p* = 0.001), occludin (*p* = 0.001), and claudin-5 (*p* = 0.024; Fig. 1). Although no significant differences in protein levels in ALS mice treated with hBM34+ cells compared with media-treated animals were found, elevated tight junction proteins were noted. In contrast, hBM-EPC treated ALS mice showed significant upregulation of ZO-1 (*p* = 0.001), occludin (*p* = 0.012), and claudin-5 (*p* = 0.039) protein levels versus media-treated ALS mice (Fig. 1). Moreover, increased ZO-1 and claudin-5 protein magnitudes in these mice were determined near their levels in control mice.

Thus, differences in tight junction protein levels in the spinal cords were detected between cell-treated and media-treated G93A SOD1 mutant mice at late disease stage (17 weeks of age). Significant downregulations of ZO-1, occludin, and claudin-5 levels were determined in media-treated ALS mice. Although increased magnitudes of all tight junction proteins were noted in ALS mice treated with hBM34+ cells, significant upregulations of analyzed proteins were established only in hBM-EPC-treated mice.

### Characteristics of pericytes in capillaries of the spinal cord

**Figure 2.**
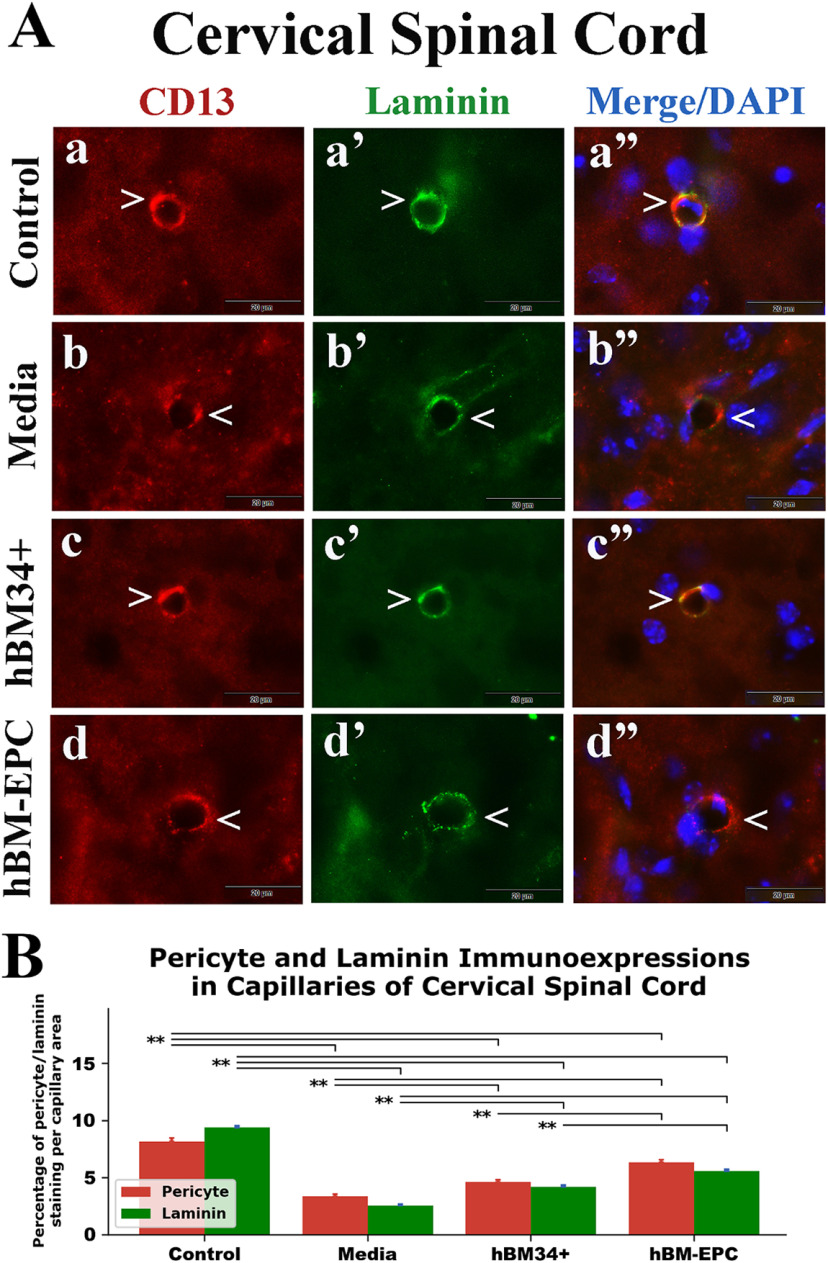
Immunohistochemical staining for pericytes (CD13) and laminin in cervical spinal cord capillaries of G93A SOD1 mice. ***A***, Double immunohistochemical staining in control mice showed high CD13 (***a***, red, arrowhead) and laminin (***a’***, green, arrowhead) immunoexpressions in cervical spinal cord capillaries. In media-treated mice, substantially decreased immunostaining of CD13 (***b***, red, arrowhead) and laminin (***b’***, green, arrowhead) in capillaries was noted. Mice treated with hBM34+ cells demonstrated higher CD13 (***c***, red, arrowhead) and laminin (***c’***, green, arrowhead) immunoexpressions in cervical spinal cord capillaries versus media-injected mice. Similarly, hBM-EPC-treated mice displayed increased immunoexpressions of CD13 (***d***, red, arrowhead) and laminin (***d’***, green, arrowhead) within capillaries of cervical spinal cords. Cervical spinal cord images (***a’’*–*d’’***) are merged with DAPI. Scale bar in all images is 20 μm. ***B***, Quantitative analysis of CD13 and laminin immunoexpressions in cervical spinal cord capillaries showed significant decreases of both antigens in media-injected mice versus controls. In hBM34+ and hBM-EPC-treated mice, significant increases of CD13 and laminin immunoexpressions were determined compared with media-treated animals. Also, hBM-EPC-treated mice displayed significantly higher antigen levels versus mice treated with hBM34+ cells; **p < 0.01.

Double immunohistochemical staining for pericytes (CD13) and laminin (basement membrane protein) was performed in the spinal cords of cell-treated, media-treated, and control animals at 17 weeks of age. Fluorescent immunoexpression for each antigen was analyzed separately in ventral horn capillaries of the cervical and lumbar spinal cord images. In the cervical spinal cord, pericyte processes and laminin appeared to fully cover capillaries in control mice (Fig. 2*Aa-a’’*), whereas capillaries in media-treated mice showed partial coverage by pericytes or laminin (Fig. 2*Ab-b’’*). Examination of fluorescence of these antigens revealed significant (*p* = 0.001) decreases of capillary pericyte and laminin immunoexpressions in media-treated ALS mice (pericyte: 3.35 ± 0.18%; laminin: 2.54 ± 0.11%) versus controls (pericyte: 8.16 ± 0.28%; laminin: 9.38 ± 0.15%; Fig. 2*B*). In contrast, many microvessels displayed increasing pericyte and laminin immunoexpressions in hBM34+ cell (Fig. 2*Ac-c’’*) and hBM-EPC-treated (Fig. 2*Ad-d’’*) mice. Significant (*p* = 0.001–0.003) increases of analyzed markers were determined in both cell-treated versus media-treated ALS mice: hBM34+ cell treated (pericyte: 4.62 ± 0.18%; laminin: 4.18 ± 0.17%); hBM-EPC treated (pericyte: 6.31 ± 0.23%; laminin: 5.57 ± 0.14%; Fig. 2*B*). Importantly, ALS mice transplanted with hBM-EPCs showed significantly (*p* = 0.001) elevated capillary pericyte/laminin immunoexpressions versus hBM34+ cell-treated mice.

**Figure 3.**
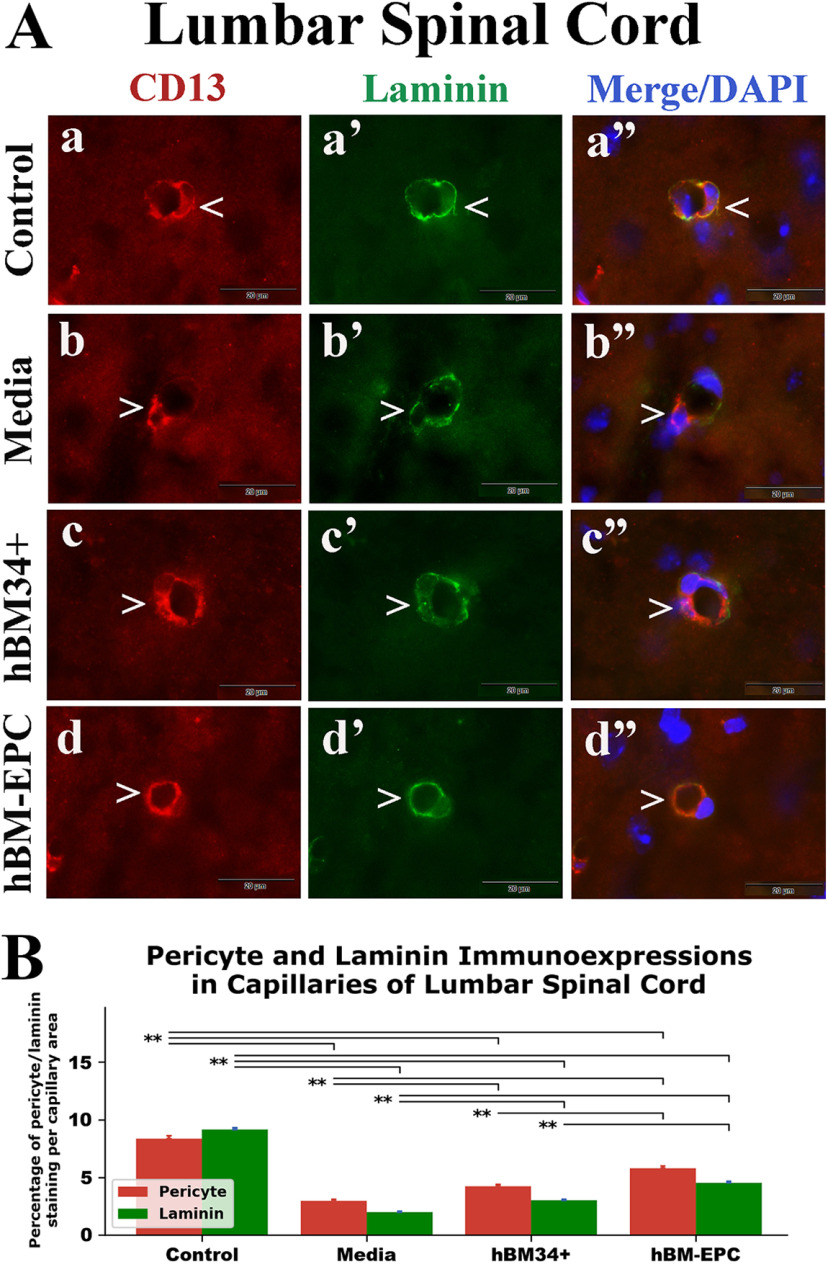
Immunohistochemical staining for pericytes (CD13) and laminin in lumbar spinal cord capillaries of G93A SOD1 mice. ***A***, Similar to the cervical spinal cord, double immunohistochemical staining in control mice showed high CD13 (***a***, red, arrowhead) and laminin (***a’***, green, arrowhead) immunoexpressions in lumbar spinal cord capillaries. A substantial decrease of immunostaining of CD13 (***b***, red, arrowhead) and laminin (***b’***, green, arrowhead) in capillaries was noted in media-treated ALS mice. Mice receiving hBM34+ cells showed higher CD13 (***c***, red, arrowhead) and laminin (***c’***, green, arrowhead) immunoexpressions in lumbar spinal cord capillaries versus media-treated mice. hBM-EPC-treated mice also displayed increased immunoexpressions of CD13 (***d***, red, arrowhead) and laminin (***d’***, green, arrowhead) within capillaries. Lumbar spinal cord images (***a’’–d’’***) are merged with DAPI. Scale bar in all images is 20 μm. ***B***, Quantitative analysis of CD13 and laminin immunoexpressions in lumbar spinal cord capillaries showed significant decreases of both antigens in media-treated mice versus controls. Immunoexpressions of CD13 and laminin significantly increased in hBM34+ and hBM-EPC treated versus media-treated mice. However, hBM-EPC-treated mice demonstrated significantly higher antigen levels versus mice treated with hBM34+ cells; **p < 0.01.

Similar to cervical spinal cord findings, control mice demonstrated lumbar spinal cord capillaries with well-defined immunostained pericytes and laminin (Fig. 3*Aa-a’’*). In media-treated animals, reduced appearance of pericytes and laminin was detected (Fig. 3*Ab-b’’*). These observations were supported by significant (*p* = 0.001) decreases of capillary pericyte and laminin immunoexpressions in media-treated ALS mice (pericyte: 2.96 ± 0.13%; laminin: 1.99 ± 0.08%) versus controls (pericyte: 8.36 ± 0.26%; laminin: 9.14 ± 0.14%; Fig. 3*B*). ALS mice treated with hBM34+ cells (Fig. 3*Ac-c’’*) or hBM-EPCs (Fig. 3*Ad-d’’*) demonstrated capillaries with abundant immunoexpressions of pericytes and laminin. Analyses of these markers showed significant (*p* = 0.001) elevations in hBM34+ cell-treated (pericyte: 4.21 ± 0.19%; laminin: 3.01 ± 0.08%) and hBM-EPC-treated (pericyte: 5.80 ± 0.20%; laminin: 4.55 ± 0.11%) mice compared with media-treated animals (Fig. 3*B*). Of note, substantially (*p* = 0.001) elevated pericyte/laminin immunoexpressions in lumbar spinal cord capillaries were determined in mice treated with hBM-EPCs versus hBM34+ cell treatment.

Together, significant decreases of pericyte and laminin immunoexpressions were established in ventral horn capillaries of cervical and lumbar spinal cords from media-treated G93A SOD1 mutant mice at 17 weeks of age compared with control animals. ALS mice treated with hBM34+ cells or hBM-EPCs showed significant increases of capillary pericyte coverage and laminin immunoexpressions in both spinal cord segments. Higher capillary pericyte/laminin immunoexpressions were determined in hBM-EPC-treated mice versus hBM34+ cell transplantation.

### Characteristics of endothelial F-actin in capillaries of the spinal cord

**Figure 4.**
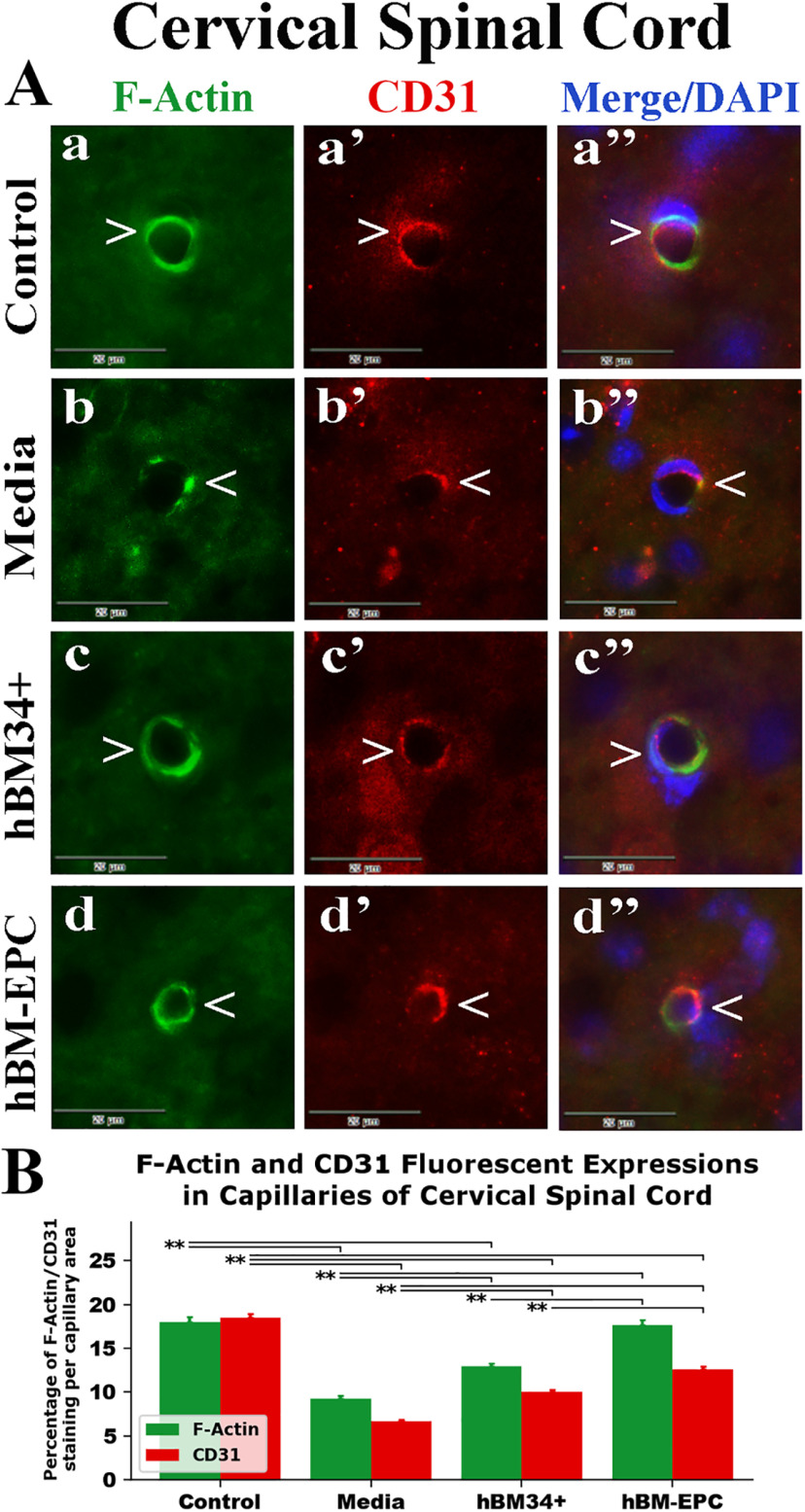
Fluorescent staining for F-actin and CD31 (PECAM-1) in cervical spinal cord capillaries of G93A SOD1 mice. ***A***, Double fluorescent staining in control mice showed continuous F-actin (***a***, green, arrowhead) and CD31 (***a’***, red, arrowhead) expressions in cervical spinal cord capillaries. In media-treated mice, substantially decreased fluorescent staining of F-actin (***b***, green, arrowhead) and CD31 (***b’***, red, arrowhead) was determined. Mice treated with hBM34+ cells demonstrated continuous F-actin (***c***, green, arrowhead) and CD31 (***c’***, red, arrowhead) expressions almost identical to controls in cervical spinal cord capillaries. Similarly, hBM-EPC-treated mice displayed near circumferential fluorescent expressions of F-actin (***d***, green, arrowhead) and CD31 (***d’***, red, arrowhead) within capillaries. Images (***a’’–d’’***) are merged with DAPI. Scale bar in all images is 20 μm. ***B***, Quantitative analysis of F-actin and CD31 fluorescent expressions in cervical spinal cord capillaries showed significant decreases of both antigens in the media-treated mice versus controls. In hBM34+ and hBM-EPC-treated mice, significant increases of F-actin and CD31 expressions were determined compared with media-treated animals. Also, hBM-EPC-treated mice displayed significantly higher antigen levels versus mice treated with hBM34+ cells; **p < 0.01.

**Figure 5.**
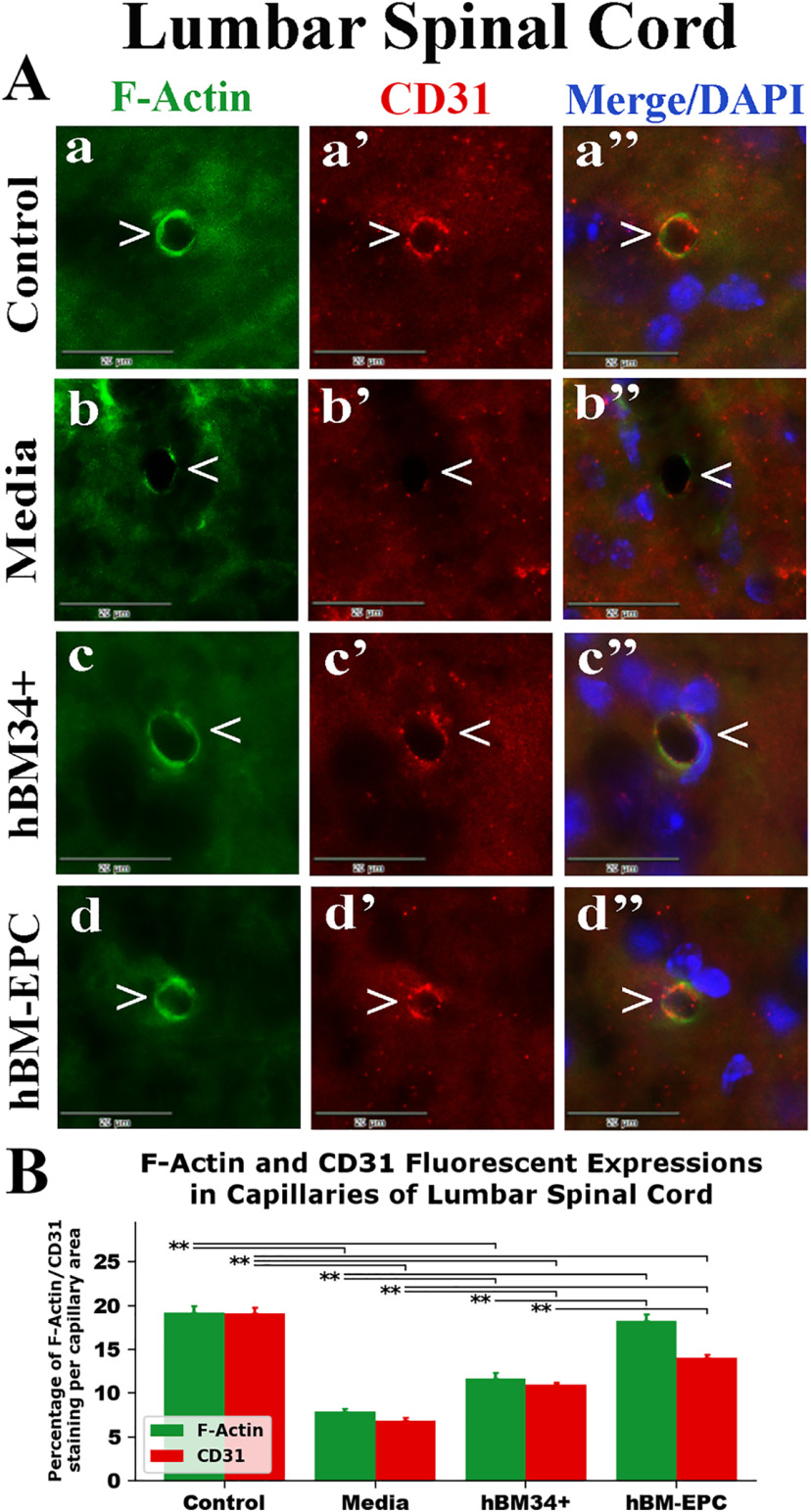
Fluorescent staining for F-actin and CD31 (PECAM-1) in lumbar spinal cord capillaries of G93A SOD1 mice. ***A***, Similar to cervical spinal cord findings, double fluorescent staining in control mice showed continuous F-actin (***a***, green, arrowhead) and CD31 (***a’***, red, arrowhead) expressions in lumbar spinal cord capillaries. A substantial decrease in fluorescent staining of F-actin (***b***, green, arrowhead) and CD31 (***b’***, red, arrowhead) was noted in media-treated mice. ALS mice treated with hBM34+ cells demonstrated continuous F-actin (***c***, green, arrowhead) and CD31 (***c’***, red, arrowhead) fluorescent expressions in lumbar spinal cord capillaries. hBM-EPC-treated mice also showed increased expressions of F-actin (***d***, green, arrowhead) and CD31 (***d’***, red, arrowhead). Images (***a’’–d’’***) are merged with DAPI. Scale bar in all images is 20 μm. ***B***, Quantitative analysis of F-actin and CD31 fluorescent expressions in lumbar spinal cord capillaries demonstrated significant decreases of both antigens in media-treated mice versus controls. Fluorescent expressions of F-actin and CD31 significantly increased in hBM34+ and hBM-EPC treated versus media-injected mice. Of note, hBM-EPC-treated mice displayed significantly higher antigen levels versus ALS mice treated with hBM34+ cells; **p < 0.01.

Fluorescent staining for F-actin (phalloidin) combined with CD31 (PECAM-1) was performed in the spinal cords of cell-treated, media-treated, and control animals at 17 weeks of age. Fluorescent expressions of F-actin and CD31 were analyzed separately in ventral horn capillaries of the cervical and lumbar spinal cord images. In control mice, F-actin and CD31 expressions were clearly presented within capillary lumen and mainly overlapped in cervical (Fig. 4*Aa-a’’*) and lumbar (Fig. 5*Aa-a’’*) spinal cords. Percentages of F-actin/CD31 fluorescent staining per capillary area were similar for these antigens in analyzed spinal cord segments: cervical (F-actin: 17.97 ± 0.63%; CD31: 18.48 ± 0.43%) and lumbar (F-actin: 19.97 ± 0.78%; CD31: 19.14 ± 0.65%) spinal cords (Figs. 4*B*, 5*B*). In media-treated mice, expressions of F-actin and CD31 were substantially reduced in capillaries of cervical (Fig. 4*Ab-b’’*) and lumbar (Fig. 5*Ab-b’’*) spinal cords. Numerous microvessels displayed partial fluorescent staining of these antigens as reflected by significant (*p* = 0.001) decreases in both cervical (F-actin: 9.24 ± 0.40%; CD31: 6.65 ± 0.22%) and lumbar (F-actin: 7.91 ± 0.33%; CD31: 6.89 ± 0.28%) spinal cords compared with controls (Figs. 4*B*, 5*B*). In contrast, ALS mice treated with hBM34+ cells or hBM-EPCs demonstrated increased fluorescent expressions of F-actin and CD31 in cervical (Fig. 4*Ac-c’’*,*d-d’’*) and lumbar (Fig. 5*Ac-c’’,d-d’’*) spinal cord capillaries.

There were distinct differences in expressions of both markers in spinal cord capillaries between cell-treated mice. In the cervical spinal cord of hBM34+ cell-treated mice, significant (*p* = 0.001) elevations of F-actin (12.92 ± 0.35%) and CD31 (10.01 ± 0.29%) were determined compared with media-treated animals (Fig. 4*B*). Similarly, capillaries in lumbar spinal cords from these cell-treated mice showed significantly increased F-actin (11.69 ± 0.63%, *p* = 0.001) and CD31 (10.98 ± 0.26%, *p* = 0.002) fluorescent expressions (Fig. 5*B*). Likewise, hBM-EPC-treated mice presented significantly (*p* = 0.001) raised expressions in cervical (F-actin: 17.69 ± 0.61% and CD31: 12.64 ± 0.32%) and lumbar (F-actin: 18.25 ± 0.82% and CD31: 14.04 ± 0.40%) spinal cord capillaries (Figs. 4*B*,5*B*). Correspondingly, significantly (*p* = 0.001) higher capillary F-actin/CD31 fluorescent expressions were determined in both cervical and lumbar spinal cords from hBM-EPC-treated mice versus hBM34+ cell treatment.

Thus, significant reductions of F-actin and CD31 expressions in the capillary lumen were determined in both cervical and lumbar spinal cord segments from media-treated G93A SOD1 mutant mice at 17 weeks of age compared with control animals. Substantial increases of these endothelial markers were established in spinal cords of ALS mice after hBM34+ cells or hBM-EPCs treatment with significantly higher F-actin/CD31 fluorescent expressions in hBM-EPC-treated mice.

## Discussion

In the present study, the effects on functional and cellular constituents of intravenously transplanted human bone marrow stem cells (hBM34+ cells or hBM-EPCs) into symptomatic G93A SOD1 mutant mice were evaluated in the spinal cord at four weeks posttransplantation in the context of potential BSCB repair. Major findings were that cell treatment effectively: (1) upregulated tight junction protein (ZO-1, occludin, and claudin-5) levels; (2) enhanced capillary pericyte coverage; (3) amended basement membrane laminin expression; and (4) improved endothelial cytoskeletal F-actin expression. Although hBM34+ cell treatment demonstrated moderate effects on analyzed constituents of BSCB, transplantation of hBM-EPCs showed more significant benefits. Here, we provided important evidence that treatment with a specific cell type derived from human bone marrow could potentially lead to BSCB repair in ALS. This cellular approach for barrier restoration, from a translational viewpoint, has advantageous therapeutic effects when initiated at the symptomatic disease stage.

The CNS microvasculature comprises an endothelial barrier controlling selective blood-tissue solute exchange for maintaining proper physiological homeostasis. The control of capillary paracellular permeability depends on inter-endothelial cell integrity via tight and adherens junctions (for review, see Bazzoni and Dejana, 2004; Dejana, 2004; Vestweber, 2008; Wallez and Huber, 2008). Downregulation of tight junction ZO-1, occludin, and claudin-5 proteins leading to microvascular leakage was reported in postmortem spinal cord tissues from sporadic and familial ALS patients (Henkel et al., 2009; Miyazaki et al., 2011). Additionally, we showed diminished level of adhesion VE-cadherin protein, associated with pervasive microvascular CNS barrier damage in ALS (Garbuzova-Davis et al., 2012). Reduced levels of tight junction proteins were also determined in animal models before motor neuron degeneration and neuroinflammation, identifying barrier breakdown as a formative event in ALS (Zhong et al., 2008; Miyazaki et al., 2011). However, decreased ZO-1 and occludin levels were shown in mainly symptomatic G93A SOD1 mutant rats (Nicaise et al., 2009). Supporting these findings, our current study demonstrated significant downregulations of ZO-1, occludin, and claudin-5 in the spinal cords of media-treated G93A SOD1 mutant mice at late disease. However, the cause of reduced tightness between adjacent ECs in ALS is still unknown. In a study by Meister et al. (2015), decreased claudin-5 and occludin in lumbar spinal cords in G93A SOD1 end-stage mice and in an *in vitro* BSCB model were shown to be associated with mutated SOD1. This might explain the state in transgenic SOD1 animal models of ALS, but not in sporadic ALS patients. Potentially, alterations in tight junction proteins are linked to EC degeneration, implicated in BBB/BSCB breakdown, as we showed in symptomatic G93A SOD1 mutant mice (Garbuzova-Davis et al., 2007a, b) and sporadic ALS patients (Garbuzova-Davis et al., 2012). Attempting to repair the BSCB barrier, our current study demonstrated significant upregulations of ZO-1, occludin, and claudin-5 in spinal cords of hBM-EPC versus hBM34+ cell-treated ALS mice. These differences suggest that restoration of endothelium integrity via replacement of damaged ECs is more effective by systemic administration of hBM-EPCs, a likelihood supported by our previous reports (Garbuzova-Davis et al., 2017b, 2018, 2019b). However, there are limitations in the present study. Determining tight junction proteins in segmental regions of the brain and spinal cord in cell-treated versus non-treated ALS mice may guide development of a cell-based therapeutic approach for restoring ALS barrier integrity. Also, examining VE-cadherin, which controls vascular permeability and leukocyte extravasation (Vestweber, 2008), may be imperative. We have planned studies to address these points.

Another focus in our current study was to validate pericyte coverage in spinal cord microvessels in cell-treated versus non-treated G93A SOD1 mutant mice. Pericytes are essential cellular components of the BBB/BSCB as discussed in (Balabanov and Dore-Duffy, 1998; Kutcher and Herman, 2009; Winkler et al., 2011). These cells have regulatory functions in CNS vascular stability, angiogenesis, microvessel permeability, and capillary blood flow. Pericytes provide functional interaction with capillary ECs via secretion of growth factors or modulation of the extracellular matrix (for review, see Allt and Lawrenson, 2001; Armulik et al., 2005). Through direct communication between pericytes and ECs, pericytes regulate capillary permeability and trans-endothelial cell transport (for review, see Edelman et al., 2006; Kamouchi et al., 2011; Geevarghese and Herman, 2014). Pericyte dysfunction is associated with various neurodegenerative disorders (Sweeney and Foldes, 2018). In ALS, pericyte status was predominantly evaluated in CNS tissues from ALS patients. Initially, we demonstrated pericyte degeneration in most capillaries from postmortem medulla and cervical/lumbar spinal cord segments via electron microscopy in sporadic ALS patients (Garbuzova-Davis et al., 2012). Later studies determined substantial reductions in pericyte number (Winkler et al., 2013) and pericyte coverage (Yamadera et al., 2015) in spinal cords of ALS patients associated with BSCB breakdown. An interesting study by Winkler et al. (2012) showed that pericyte capillary coverage and ZO-1/occludin were reduced in spinal cord segments compared with brain regions in wild-type mice. The authors also demonstrated additional pericyte reductions and decreases of tight junction proteins in spinal cord capillaries of pericyte-deficient mutant mice, confirming the role of pericytes in BSCB alterations. However, to date there are no data regarding CNS capillary pericytes status in animal models of ALS at any disease stage. Although one report (Coatti et al., 2017) demonstrated that weekly intraperitoneal injections of human adipose-derived pericytes into presymptomatic G93A SOD1 mutant mice significantly extended survival of only males and stimulated expression of antioxidant enzymes in brains and spinal cords of end-stage animals. Nevertheless, no evidence has been provided regarding pericyte capillary coverage in the CNS tissues of ALS mice *before* or *after* cell treatment. Moreover, human RNA was not identified in the CNS of ALS mice treated with human derived pericytes by Coatti et al. (2017). In this context, our recent study (Garbuzova-Davis et al., 2021) revealed transplanted human bone marrow stem cells engraftment into CNS capillaries of symptomatic ALS mice via detection of endothelial cell-associated human DNA. Our results showed greater concentrations of human DNA in mice receiving hBM-EPCs versus hBM34^+^ cells, potentially because of replacement of damaged CNS ECs in ALS mice. Since disruption of intercellular communications between BBB/BSCB components may be an issue in ALS, reestablishing crosstalk between pericytes and endothelial cells is a potential target for restorative therapies (Geevarghese and Herman, 2014). In our current study, we demonstrated for the first time a substantial decrease of pericytes in spinal cord capillaries of media-treated late symptomatic G93A SOD1 mutant mice and a significant increase of capillary pericyte coverage mainly in ALS mice treated with hBM-EPCs.

Additionally, a significant decrease of laminin (major non-collagenous basement membrane glycoprotein) was determined in capillaries of cervical and lumbar spinal cords from media-treated G93A SOD1 mutant mice at 17 weeks of age compared with control animals. These results support our previous study showing marked reductions of laminin expression in spinal cord capillaries of ALS mice at early and late disease stages (Garbuzova-Davis et al., 2007b), suggesting disruption of vascular basement membrane integrity. A substantial increase of laminin immunoexpression was established in cervical/lumbar spinal cord segments after cell treatment with higher levels in hBM-EPC-treated mice versus hBM34+ cell transplantation. Since the vascular basement membrane interacts directly with pericytes and endothelial cells (Timpl, 1989; Paulsson, 1992), restoring this basement membrane protein is potentially linked to functional BSCB repair. However, in addition to laminin, other basement membrane components such as collagen IV and/or heparan-sulfate proteoglycans should be investigated.

Finally, F-actin was examined in capillary endothelium of the spinal cords in cell-treated versus media-treated G93A SOD1 mutant mice. Actin has an essential role in cell motility and morphology by cytosolic organelle stabilization (Zigmond, 2004; Fletcher and Mullins, 2010; Dominguez and Holmes, 2011). Actin is comprised of monomeric globular actin (G-actin), which polymerizes to F-actin (Kozuka et al., 2007; Oda and Maéda, 2010). Cytoskeletal F-actin maintains connections between adjacent cells via tight and adherens junctions (Itoh et al., 1997; Fanning et al., 1998; Waschke et al., 2005; Odenwald et al., 2017; Sakakibara et al., 2018). F-actin interacts with the tight junction proteins ZO-1 and occludin (Fanning et al., 1998; Odenwald et al., 2017), proteins implicated in endothelial barrier integrity. Our current study demonstrated for the first time significant reductions of F-actin expression in capillary lumen in both cervical and lumbar spinal cord segments from media-treated G93A SOD1 mutant mice at late stage of disease. Diminished endothelial F-actin expressions in these ALS mice were detected concurrently with downregulations of tight junction proteins in the spinal cords, supporting interaction of F-actin with tight junction proteins. However, it is still unclear whether F-actin dysregulation is linked to EC degeneration in ALS. Potentially this cytoskeletal protein initiates endothelial cell damage by re-organization of filamentous strips in cytosol in response to external harmful blood-derived substances. Also, F-actin levels may reflect cell apoptosis (Suarez-Huerta et al., 2000; Desouza et al., 2012). However, these possibilities need clarification. Regardless of F-actin’s role in ALS EC degeneration, substantially increased F-actin fluorescent expression was detected in spinal cord capillaries mainly in hBM-EPC-treated mice. Our cell treatment effect may be related to replacement of damaged ECs with “healthy” hBM-EPCs, leading to microvascular repair in ALS. Thus, endothelial cytoskeletal F-actin may indicate endothelium status.

Supportive evidence for the potent replacement of damaged ECs via hBM-EPC transplant may be found in results of a study in another disease model such as ischemic stroke (Garbuzova-Davis et al., 2017a). Study findings showed that intravenous administration of hBM-EPCs prelabeled with β-galactosidase into ischemic stroke rats substantially repaired the altered BBB via robust cell engraftment within numerous brain capillaries. In addition to specific outcomes of hBM-EPCs toward restoration of the compromised blood-CNS-barrier, hBM-derived stromal cells (hBM-SCs) per se may exert neuroprotective effects. For example, a meta-analysis of 27 studies using a Parkinson’s disease (PD) rodent model demonstrated that naive hBM-SC therapy increased dopaminergic neurons and improved behavioral deficits (Liu et al., 2020). Moreover, a clinical pilot study showed safety and efficacy of bilateral transplantation of hBM-derived mesenchymal stem cells (hBM-MSCs) into the subventricular zone of PD patients (Venkataramana et al., 2012). Also, a recent report (Schiess et al., 2021) demonstrated safety and tolerability of a single intravenous hBM-MSC infusion in PD patients. Thus, depending on the administered cell type derived from bone marrow (EPCs or mesenchymal/stromal stem cells), different therapeutic achievement can be expected.

In conclusion, we previously demonstrated that systemic administration of hBM-EPCs versus hBM34+ cells into symptomatic G93A SOD1 mutant mice more effectively restores barrier integrity in the CNS, leading to improved behavioral disease outcomes and delayed disease progression. Beneficial effects of hBM-EPCs transplantation may be because of cell engraftment into capillary lumen within the spinal cord and brain, resulting in substantially restored capillary ultrastructure, significantly decreased capillary permeability, and maintained motor neuron survival. Thus, one potential administered cell action is replacement of damaged ECs within mouse CNS capillaries. In addition, the current study demonstrated improvements of hBM-EPCs versus hBM34+ cells on BSCB constituents such as tightness between ECs, capillary pericyte coverage, basement membrane laminin immunoexpressions, and endothelial cytoskeletal F-actin fluorescent expressions four weeks after cell transplant. The likely reason for the noted effectiveness of hBM-EPCs is that these specialized cells are more prone to develop into ECs than the more generic hematopoietic hBM34+ stem cells. Altogether, there is strong evidence that treatment with this specific cell type derived from human bone marrow could possibly lead to BSCB repair in ALS. Also, systemic cell transplantation may be considered a preferable route of administration for clinical ALS applications intended to restore barrier integrity.

## Synthesis

Reviewing Editor: Christopher Colwell, UCLA School of Medicine Decisions are customarily a result of the Reviewing Editor and the peer reviewers coming together and discussing their recommendations until a consensus is reached. When revisions are invited, a fact-based synthesis statement explaining their decision and outlining what is needed to prepare a revision will be listed below. The following reviewer(s) agreed to reveal their identity: Krishna Aluri. Note: If this manuscript was transferred from JNeurosci and a decision was made to accept the manuscript without peer review, a brief statement to this effect will instead be what is listed below.

Synthesis: This study demonstrates a solid mechanism by which human bone marrow endothelial progenitor cells improve the functionality of the blood-spinal cord barrier in a rodent model for amyotrophic lateral sclerosis. This information is important in understanding the relevance of the BSCB in motor neuron diseases and identifies a novel target for therapeutics development. Overall, we found the work to be excellent with a manuscript that is well written and supported by clear figures.

Reviewer's comments:

1. In the Result section (pg. 11), the relative quantification of target protein levels does not to be stated in the text as they are included in Figure 1. The use of (presumably) a dash before each value is confused in that it may represent a negative sign to some readers.

2. The term “immunoexpression” is not correct when describing F-actin staining as phalloidin is not an antibody.

3. The authors showed data for a dose of 1x106 cells/mouse, we understand that this dose was selected based on highest dose from their CD34+ cells study previously published. Did they evaluate any other doses using hBM-EPCs?

4. In the discussion, the authors cited their previous work using hBM-EPCs delayed ALS progression and motor neuron survival in in ALS mice. Can the authors include quantitative numbers compared to controls here, so readers can gauge the impact of such treatment and compare it with other therapeutic strategies?

5. Please include few sentences in discussion reviewing and citing the work done using hBMCs in other disease areas (ex. Parkinson's disease) and how this strategy can have a broader impact on neuroscience
